# Clinical Evaluation of a New Molecular Test for the Detection of Organisms Causing Vaginitis and Vaginosis

**DOI:** 10.1128/jcm.01748-22

**Published:** 2023-02-28

**Authors:** Rebecca A. Lillis, R. Lamar Parker, Ronald Ackerman, Jamie Ackerman, Stephen Young, Alice Weissfeld, Ernest Trevino, Irving Nachamkin, LaShonda Crane, Jacqueline Brown, Christina Huang, Xiaohong Liu, Barbara Van Der Pol

**Affiliations:** a Department of Medicine, Louisiana State University Health Sciences Center, New Orleans, Louisiana, USA; b Unified Women’s Clinical Research, Raleigh, North Carolina, USA; c Comprehensive Clinical Research, West Palm Beach, Florida, USA; d TriCore Reference Laboratories, University of New Mexico HSC, Albuquerque, New Mexico; e Microbiology Specialists Inc., Houston, Texas, USA; f Perlman School of Medicine, University of Pennsylvania, Philadelphia, Pennsylvania, USA; g Planned Parenthood Gulf Coast Inc., Houston, Texas, USA; h Clinical Trials Network, Houston, Texas, USA; i Cepheid Inc., Sunnyvale, California, USA; j Heersink School of Medicine, University of Alabama Birmingham, Birmingham, Alabama, USA; Marquette University

**Keywords:** bacterial vaginosis, *Candida vaginitis*, point of care test, *Trichomonas vaginalis*, molecular diagnostics, vaginitis diagnosis panel

## Abstract

In this prospective, observational, method comparison clinical study, the Xpert Xpress MVP test (MVP) was evaluated using both clinician-collected (CVS) and self-collected vaginal swabs (SVS) collected in a clinical setting. The study was conducted at 12 sites, including point-of-care (POC) settings, from geographically diverse locations in the United States. Participants were biologically female patients ≥ 14 years old with signs and/or symptoms of vaginitis/vaginosis. MVP test results for BV were compared to the BD MAX Vaginal Panel (BDVP). Results for Candida group and Candida glabrata and Candida krusei targets (species not differentiated) were assessed relative to yeast culture followed by mass spectrometry for species identification. Trichomonas vaginalis (TV) results were compared relative to a composite method that included results from the BDVP and InPouch TV culture. The investigational test demonstrated high positive percent agreement ranging from 93.6 to 99.0%, and negative percent agreement ranging from 92.1% to 99.8% for both CVS and SVS specimens, indicating it may be a valuable tool for the diagnosis of vaginitis/vaginosis in laboratory and POC settings.

## INTRODUCTION

Vaginal complaints are the most common reason for women to seek medical care, with global prevalence ranging from 23% to 29% in women of reproductive age (1). Most women will experience at least one episode of vaginitis in their lifetime (2, 3). Vaginal complaints are most often a result of either bacterial vaginosis (BV), vulvovaginal candidiasis (VVC), or trichomoniasis. BV is the most common cause of vaginal discharge in reproductive aged women, accounting for 40 to 50% of cases (4). Among women aged 14 to 49 participating in the National Health and Nutrition Examination Survey (5), the prevalence of BV was found to be 29.2%, and the estimated annual incidence of trichomoniasis among women in the US is 3.5 million cases (6).

While vaginitis and vaginosis are common, achieving an accurate diagnosis is elusive in most practice settings. Practice guidelines recommend the use the Amsel’s clinical criteria (7), the Nugent score (8), or culture-based methods for diagnosis of symptomatic vaginitis/vaginosis (9, 10). According to Amsel criteria, diagnosis of BV is based upon the presence of 3 out of 4 of the following clinical criteria: (i) vaginal pH > 4.5; (ii) homogenous white/gray adherent vaginal discharge; (iii) the presence of clue cells (vaginal epithelial cells covered in bacteria); and (iv) a positive whiff test (fishy odor after addition of potassium hydroxide) (7). Although frequently used as an office-based assessment, the Amsel criteria is reported to be subjective, and prone to high rate of misdiagnosis (11). Further, given the reduction in frequency of speculum-assisted examinations (12), exacerbated in response to the SARS-COV-2 pandemic reduction in clinical services for sexually transmitted infections (STI), Amsel criteria cannot be fully evaluated, and rely on only 3 rather than 4 criteria, which further reduces accuracy. While Nugent score (a Gram stain scoring system based on the quantitative assessment of *Lactobacillus*, *Gardnerella*, and *Mobiluncus* spp.) is deemed less subjective, it is dependent on user’s expertise with microscopy, has higher turnaround time, and is prone to misdiagnosis, especially for fungal-based vaginosis and mixed infections (11). This method is not widely used in the clinical setting. In order to perform these tests successfully, the clinician must have access to microscopy, proficiency in microscopy, pH paper, and the other supplies needed to perform a wet mount and KOH screen.

A recent study found that, in 1 health care system, less than 25% of women presenting with symptoms of vaginitis/vaginosis were evaluated with any of these point-of-care (POC) tests (13). Even when used properly, these tests often do not lead to an accurate diagnosis of vaginosis. Published studies demonstrate that nucleic acid amplification tests (NAAT) are more sensitive and specific than point-of-care (POC) microscopy for the detection of organisms associated with vaginitis and vaginosis (14). The accuracy of microscopy alone for the diagnosis of trichomoniasis is poor (15), and thus, for nearly a decade, the Centers for Disease Control and Prevention (CDC) has recommended NAAT for diagnosis of trichomoniasis in lieu of other testing strategies. Further complicating the diagnosis of vaginitis and vaginosis is the high frequency of co-infections, occurring in in an estimated 10 to 20% of women (13, 16, 17).

The Xpert Xpress MVP (MVP; Cepheid) test is designed to be an automated, qualitative *in vitro* diagnostic PCR test for the detection of DNA targets from anaerobic bacteria associated with BV, *Candida* species associated with VVC, and Trichomonas vaginalis (TV). MVP testing is performed using both the Cepheid GeneXpert Dx System and the GeneXpert Xpress System, which allows an untrained operator to run a test by performing four simple steps: (i) mixing the specimen, (ii) transferring the liquid sample to the cartridge with a transfer pipette, (iii) running the test, and (iv) viewing the results.

## MATERIALS AND METHODS

### Study design and setting.

This was a multicenter, prospective, cross-sectional, clinical study to evaluate the diagnostic accuracy of MVP. Participants were enrolled from 12 sites across the United States between March and October 2020. Most sites enrolled only participants 18 years of age and older. Of the 12 sites, 10 participated in specimen collection and MVP testing, 2 sites participated in specimen collection only. Of the 10 MVP testing sites, 1 was a laboratory with routine clinical laboratory testing capabilities that tested patient specimens either collected from the same location or received from collection-only sites. All testing performed at the laboratory site was done on the GeneXpert Dx instrument by trained operators (trained users). The other 9 testing sites were POC environments where testing was performed outside the clinical laboratory setting, near to or at the side of the patient, including family planning/sexual health clinics, OB/GYN clinics, and women’s health clinics. Testing at 8 of the POC sites was performed on the GeneXpert Xpress System by non-laboratory health care personnel with no experience, using either the Cepheid CLIA-waived or moderately complex tests (untrained users). The protocol was reviewed and approved by each site’s institutional review board.

### Participants and specimens.

A convenience sample of consecutive clinic attendees meeting the eligibility criteria were included until study enrollment was complete. Study participants were biological females ≥ 14 years old who met all of the following inclusion criteria: (i) provided documented informed consent (or assent if participants were minors), and (ii) presented with signs and/or symptoms of vaginitis/vaginosis, which included the following: abnormal vaginal discharge; dysuria; vulvar/vaginal itching, burning, irritation, pain or vulvar edema; coital pain; and/or vaginal odor. Study participants who were previously enrolled into the study were excluded.

**FIG 1:**
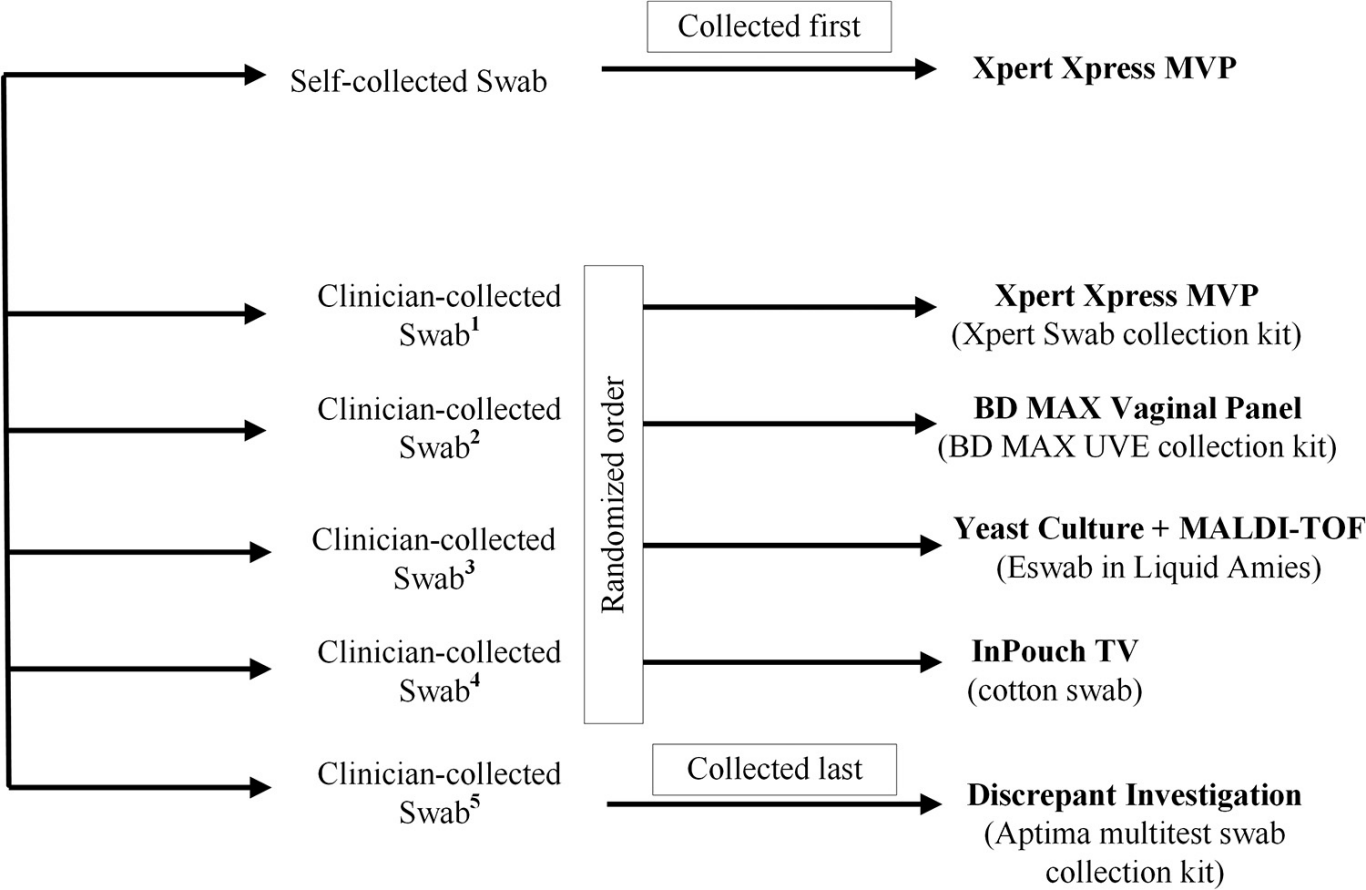
Swab Collection and Testing Workflow. One self-collected swab (SVS) was always collected first; five clinician-collected swabs (CVS) were collected, where the first 4 (Swabs 1 to 4) were collected in a randomized order; and 1 CVS (Swab 5) was always collected last and tested as needed for discrepant result investigation.

An overview of the study workflow is presented in Fig. 1. Study participants obtained 1 self-collected vaginal swab (SVS) in a clinical setting and provided 5 clinician-collected vaginal swab (CVS) specimens. The SVS was always the first swab collected with the Cepheid Xpert Swab Specimen Collection Kit for MVP testing. Of the 5 CVS, the first 4 swabs were collected in a randomized order: ESwab in Liquid Amies for yeast culture followed by MALDI-TOF confirmation, BD MAX UVE Specimen Collection Kit for BD MAX Vaginal Panel (BDVP; BD Diagnostics), Cepheid Xpert Swab Specimen Collection Kit for MVP testing, and a cotton swab for InPouch TV culture (TV Culture; Biomed Diagnostics Inc.). The 5^th^ CVS was collected with the Aptima Multitest Swab Specimen Collection Kit (Hologic) for testing, as needed, to investigate discrepant results. All specimens were shipped to reference laboratories for comparator testing within a day of collection, and were stored at the temperatures recommended by the respective manufacturer’s package insert until testing was complete.

### MVP and comparator methods.

The MVP test has 4 reportable results for BV (determined by a proprietary algorithm based on 3 DNA targets), Candida group (including C. albicans, C. tropicalis, C. parapsilosis, and C. dubliniensis), Candida glab-krus, and TV. Performance of MVP was determined relative to comparator methods as presented in Table 1.

**TABLE 1:** Performance evaluation of the Xpert Xpress MVP test

Xpert Xpress MVP test result	BV^*a*^	Candida group^*e*^/Candida glab-krus	TV
Comparator method	BDVP^*b*^	Yeast culture + MALDI-TOF	BDVP + TV culture
Discrepant resolution	ABV^*c*^	BDVP	ATV^*d*^

a BV, bacterial vaginosis.

b BDVP, BD MAX Vaginal Panel.

c ABV, Aptima BV.

d ATV, Aptima TV.

e Includes C. albicans, C. tropicalis, C. parapsilosis, and C. dubliniensis.

Specifically, MVP BV results were assessed head to head with the BD MAX Vaginal Panel (BDVP; BD Diagnostics) Candida group (Candida albicans, Candida tropicalis, Candida parapsilosis, and Candida dubliniensis), and Candida glab-krus (Candida glabrata and Candida krusei) results were assessed relative to the reference standard of yeast culture, followed by testing of a colony with morphology consistent with Candida by MALDI-TOF (Bruker Daltonics) for species identification. Due to the low number of Candida glabrata and Candida krusei in clinical specimens, contrived specimens were included in the analysis. MVP TV results were assessed relative to a composite method, where a positive result from either BDVP or the InPouch TV culture meant the specimen was from a positive participant, while negative results from both tests meant the participant was negative. All comparator testing was performed according to the respective manufacturers’ instructions.

Specimens for which MVP and comparator method test results were not in agreement underwent additional testing with the discrepant test methods: Aptima BV (ABV) for BV results, BDVP for Candida results, and Aptima TV (ATV) for TV results (Hologic). Testing to investigate discrepant results was performed according to the manufacturer’s instructions.

Specimen preparation and testing were conducted according to instructions in the protocol or comparator package insert. MVP testing was performed within 24 h following collection at POC sites, and within 5 days following collection at the laboratory site. If the initial test result was indeterminate, a single retest was performed if enough specimen volume remained. If an indeterminate result was obtained for the second test, no additional testing was performed. Results were for study purposes only and not used for patient management.

### Statistical methods.

Performance was determined based on concordance of the MVP test for each result relative to the comparator methods. Though Candida results were assessed relative to the reference standard and diagnostic accuracy could be presented in terms of sensitivity and specificity, positive percent agreement (PPA) and negative percent agreement (NPA) were used for ease of interpretation alongside BV and TV results. Point estimates for PPA and NPA were calculated with corresponding 95% confidence interval (CI) using the Wilson Score method. For each reportable result, analyses were performed separately, and results from CVS specimens were compared to results from SVS specimens in order to evaluate equivalence between specimen collection methods. A McNemar’s test was conducted to evaluate potential difference in specimen collection methods. User training status was used as a factor in subgroup analysis for poolability using Fisher’s exact test. For both statistical tests, *P* value > 0.05 indicated no statistically significant differences. All statistical analyses were performed using R studio (2020 [1.3.1093-1, Apricot Nasturtium]).

## RESULTS

### Study specimens.

**FIG 2:**
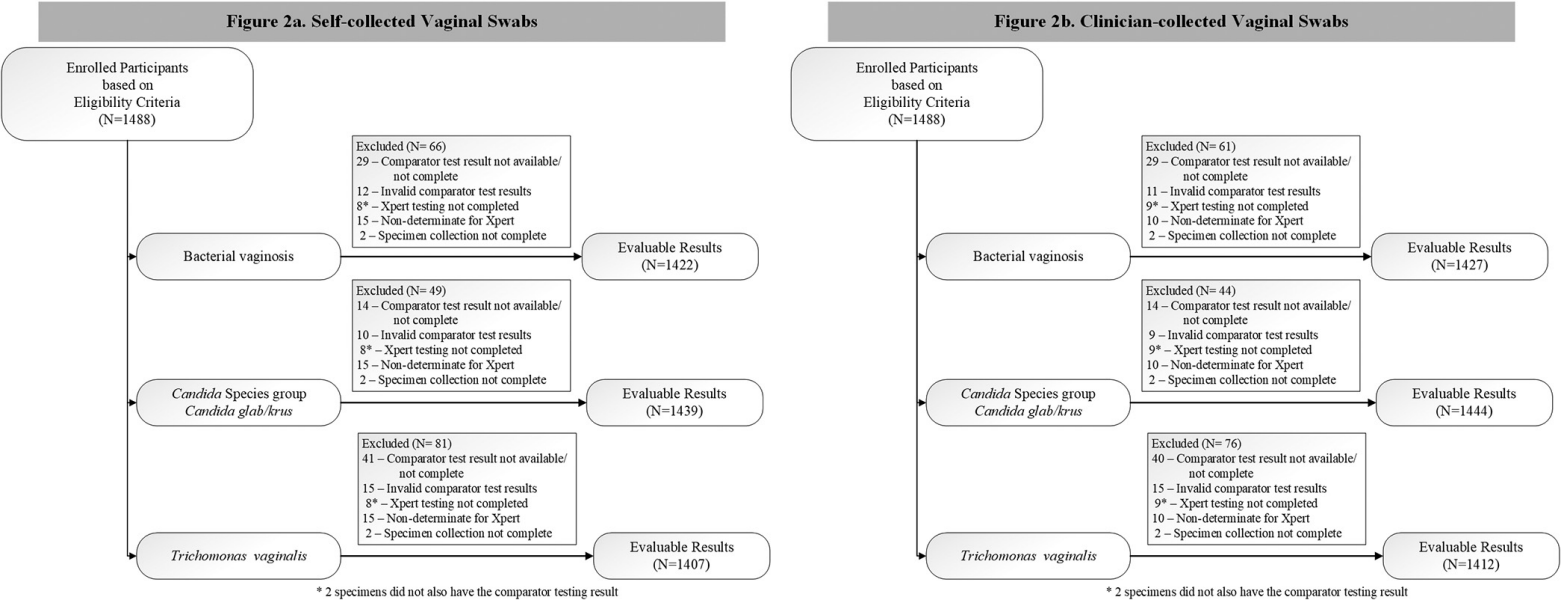
Disposition of Enrolled Participants for Self-collected Vaginal Swabs (2a) and Clinician-collected Vaginal Swabs (2b). Reasons for exclusion included unavailable/incomplete comparator test results, invalid comparator test results, incomplete Xpert test results, non-determinate Xpert test results, and incomplete specimen collection.

We enrolled 1488 participants in the study. Of these, 1478 participants were evaluable for at least 1 of the 4 MVP reportable results. The 3 main reasons specimens were excluded from analyses included unavailable, invalid, or incomplete comparator test results and indeterminate MVP results. The evaluable study population included specimens from 1422 participants for BV, 1439 for Candida results, and 1407 for TV (Fig. 2).

Demographics of the 1478 participants who provided specimens included in the final data sets used for the analyses are presented in Table 2. A large number of participants (38.9%) were between ages 18 and 29 years. The majority of women enrolled were white (56.4%), and Black or African American (39.2%). Half (50.0%) of the women reported a history of BV. Only 7.4% women reported pregnancy at the time of enrollment.

**TABLE 2:** Demographic and clinical characteristics of evaluable subjects

Category	*N*	% (*N* = 1478)
Age group (yrs)		
14 to 17	2	0.1%
18 to 29	575	38.9%
30 to 39	403	27.3%
40 to 49	233	15.8%
≥50	265	17.9%
Race		
White	834	56.4%
Black or African American	580	39.2%
Asian	22	1.5%
American Indian or Alaska native	11	0.7%
Native Hawaiian or other Pacific Islander	3	0.2%
Mixed/Unknown	28	1.9%
Ethnicity		
Hispanic or Latino	223	15.1%
Not Hispanic or Latino	1255	84.9%
Baseline clinical characteristics		
Pregnant	109	7.4%
With menses at enrollment	95	6.4%
Using anti-fungals in ≤24 hours	53	3.6%
Using antibiotics in ≤24 hours	24	1.6%
Using estrogen therapy in ≤24 hours	21	1.4%
Prior history of BV	739	50.0%
With intercourse in ≤24 hours	86	5.8%

### Clinical performance.

**(i) Overall MVP performance.** As presented in Table 3, MVP demonstrated PPA and NPA of 93.8% for BV detection in CVS specimens, and 94.0% and 92.9% in SVS specimens, respectively. For *Candida* spp. Group detection, PPA and NPA was 98.0% and 94.6% in CVS specimens, and 97.5% and 92.1% in SVS specimens, respectively. MVP demonstrated PPA and NPA of 97.3% and 99.6% for Candida glab-krus detection in fresh and contrived CVS specimens, respectively, and 98.6% and 99.3% in SVS specimens, respectively. For TV detection, PPA and NPA in CVS specimens was 97.3% and 99.6%, respectively, and 97.3% and 99.8% in SVS specimens, respectively. Prevalence of BV, VVC, and TV single infection rates were similar for clinician- and patient-collected vaginal swabs for the MVP assay.

**TABLE 3:** Overall Performance of MVP

	Clinician-collected (CVS)				Self-collected (SVS)			
	PPA		NPA		PPA		NPA	
Pathogen	n/N	% (95% CI)	n/N	% (95% CI)	n/N	% (95% CI)	n/N	% (95% CI)
BV	531/566^*a*^	93.8 (91.5%–95.5%)	808/861^*b*^	93.8 (92.0%–95.3%)	533/567^*c*^	94.0 (91.7%–95.7%)	794/855^*d*^	92.9 (90.9%–94.4%)
*Candida* spp. group^*q*^	396/404^*e*^	98.0 (96.1%–99.0%)	984/1040^*f*^	94.6 (93.1%–95.8%)	393/403^*g*^	97.5 (95.5%–98.7%)	954/1036^*h*^	92.1 (90.3%–93.6%)
Candida glab-krus (fresh prospective)	44/47^*i*^	93.6 (82.8%–97.8%)	1392/1397^*j*^	99.6 (99.2%–99.9%)	45/46^*k*^	97.8 (88.7%–99.6%)	1384/1393^*l*^	99.3 (98.8%–99.7%)
Candida glab-krus (contrived^*r*^)	98/99	99.0 (94.5%–99.8%)	27/28	96.4 (82.3%–99.4%)		N/A		N/A
TV	73/75^*m*^	97.3 (90.8%–99.3%)	1332/1337^*n*^	99.6 (99.1%–99.8%)	72/74^*o*^	97.3 (90.7%–99.3%)	1330/1333^*P*^	99.8 (99.3%–99.9%)

a Of the 35 false negative results, 14 were also negative and 21 were positive by Aptima BV assay.

b Of the 53 false positive results, 25 were also positive and 28 were negative by Aptima BV assay.

c Of the 34 false negative results, 12 were also negative and 22 were positive by Aptima BV assay.

d Of the 61 false positive results, 23 were also positive and 38 were negative by Aptima BV assay.

e Of the 8 false negative results, 5 were also negative and 3 were positive by BD MAX assay.

f Of the 56 false positive results, 31 were also positive and 24 were negative by BD MAX assay; 1 no result.

g Of the 10 false negative results, 5 were also negative and 5 were positive by BD MAX assay.

h Of the 82 false positive results, 38 were also positive and 43 were negative by BD MAX assay; 1 no result.

i Of the 3 false negative results, 2 were also negative and 1 was positive by BD MAX assay.

j Of the 5 false positive results, all 5 were negative by BD MAX assay.

k Of the 1 false negative result, 1 was also negative by BD MAX assay.

l Of the 9 false positive results, all 9 were negative by BD MAX assay.

m Of the 2 false negative results, 1 was negative and 1 was positive by Aptima TV assay.

n Of the 5 false positive results, 4 were positive by Aptima TV assay; 1 no result.

o Of the 2 false negative results, 1 was negative and 1 was positive by Aptima TV assay.

p Of the 3 false positive results, all 3 were positive by Aptima TV assay.

q Includes C. albicans, C. tropicalis, C. parapsilosis, and C. dubliniensis.

r Contrived specimens were prepared using individual negative clinical CVS and SVS specimens.

### Single and multiple infection rates.

**FIG 3:**
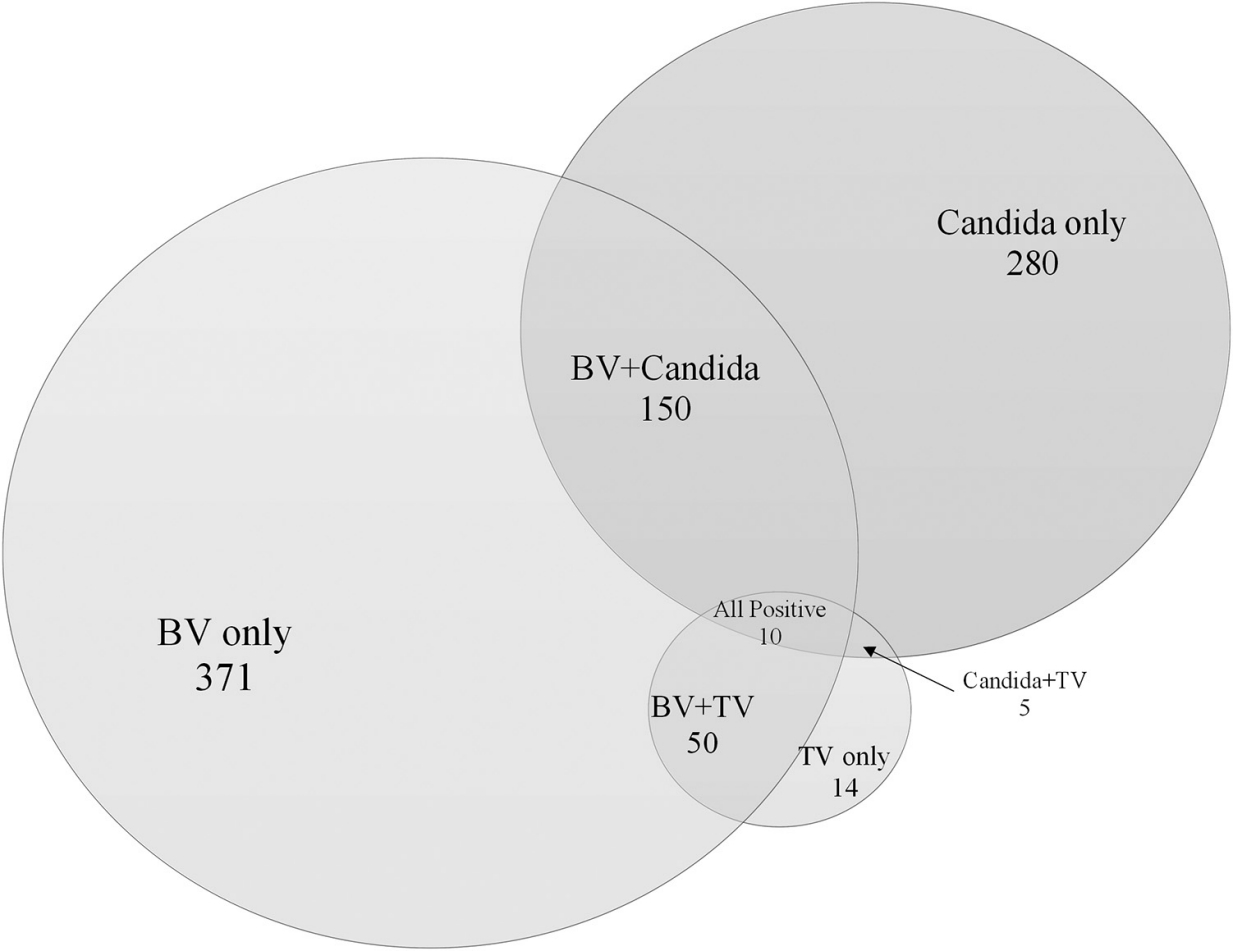
Single and Multi-infections based on Comparator Method Rates of infection with BV, TV, and VVC (all Candida isolates pooled) in a proportional Venn diagram.

Table 4 and Fig. 3 summarize the single and multiple infection rates in the evaluable study population based on the comparator method. Overall, multiple infection was observed in 17.0% and 17.9% of CVS and SVS specimens, respectively. The most prevalent multiple infection detected by MVP in CVS and SVS specimens was a combination of BV and *Candida* spp. group (11.0% and 11.5%, respectively), followed by a combination of BV and TV (3.5% for both).

**TABLE 4:** Single and multi-infection rates based on comparator method and MVP

Analytes detected	Comparator method (*N* = 1446)	By MVP	
		Clinician-collected (*N* = 1473)	Self-collected (*N* = 1474)
All negative	39.1% (566)	37.1% (546)	35.4% (522)
BV^*a*^ only	25.7% (371)	24.4% (360)	24.4% (359)
BV, *Candida* spp. group	9.7% (140)	11.0% (162)	11.5% (170)
BV, Candida glab-krus	0.4% (5)	0.5% (7)	0.5% (7)
BV, *Candida* spp. group, Candida glab-krus	0.4% (5)	0.3% (5)	0.7% (10)
BV, *Candida* spp. group, TV^*b*^	0.7% (10)	0.9% (13)	0.8% (12)
BV, TV	3.5% (50)	3.5% (52)	3.5% (52)
*Candida* spp. group only	16.9% (244)	18.3% (269)	18.9% (278)
*Candida* spp. group, Candida glab-krus	0.1% (2)	0.3% (5)	0.5% (8)
*Candida* spp. group, TV	0.3% (4)	0.3% (4)	0.3% (4)
*Candida* spp. group, Candida glab-krus, TV	N/A	0.1% (1)	N/A
Candida glab-krus only	2.4% (34)	2.0% (30)	1.9% (28)
Candida glab-krus, TV	0.1% (1)	0.1% (1)	0.1% (1)
TV only	1.0% (14)	0.7% (10)	0.6% (9)
Total multiple infections	18.7% (271)	17.0% (250)	17.9% (264)

a BV, bacterial vaginosis.

b TV, Trichomonas vaginalis.

**(i) Equivalence between specimen collection methods.** There were no statistically significant differences in performance between CVS and SVS specimens for BV, Candida glab-krus, and TV results of the MVP test (*P* = 0.1599, 0.1088, and 0.1573, respectively). More positive Candida group results were called among SVS relative to CVS specimens (*P* = 0.0052). However, the difference is not considered clinically meaningful (98.0% versus 97.5% sensitivity, and 94.6% versus 92.1% specificity), and it could be explained by the order in which specimens were collected, since the SVS specimen were always collected first while the order of the CVS collection was randomized, and potentially resulted the CVS Xpert swab obtained after 3 other collections. Alternatively, the self-obtained specimen may perform incrementally better, as has been well described for chlamydia and gonorrhea testing (18–20).

**(ii) Performance of test by user type.** The study design incorporated both laboratory-based testing by trained users and POC testing by untrained users. Results from Fisher’s exact test demonstrated the clinical performance was consistent across users, regardless of training status (Table 5).

**TABLE 5:** Performance of MVP by user type

MVP result	Positive percent agreement			Negative percent agreement		
	Trained users	Untrained users	*P* value	Trained users	Untrained users	*P* value
BV^*a*^	95.7% (337/352)	93.1% (727/781)	0.1064	93.8% (375/400)	93.2% (1227/1316)	0.8187
Candida group	97.0% (161/166)	98.0% (628/641)	0.3913	92.7% (545/588)	93.6% (1393/1488)	0.4357
Candida glab-krus	94.7% (36/38)	96.4% (53/55)	1.0000	98.9% (708/716)	99.7% (2068/2074)	0.0120
TV^*b*^	97.1% (66/68)	97.5% (79/81)	1.0000	100% (682/682)	99.6% (1980/1988)	0.2153

a BV, bacterial vaginosis.

b TV, Trichomonas vaginalis.

## DISCUSSION

In this study, MVP demonstrated a high PPA and NPA relative to the comparator methods, providing results for all 3 causes of vaginitis/vaginosis from a single swab specimen in both laboratory-based and POC settings. Both the patient self-collected and clinician-collected vaginal swabs performed similarly for all results of the test in the hands of trained and untrained users alike.

Diagnosis of vaginitis/vaginosis is most commonly based on Amsel criteria, and less often on Nugent score and/or yeast culture (9, 10). Unfortunately, these methods are highly dependent on the user performing the assessment and have low accuracy (21). NAATs for BV and TV diagnosis in symptomatic women are recommended for their diagnostic accuracy and ease of use (22, 23). Since these tests have not previously been available in the POC setting clinicians have relied on older, less reliable, methods of diagnosis. Hillier et al., in a recent study investigating the diagnostic algorithms used by clinicians for women with vaginitis, found that CDC-recommended POC testing was infrequently performed. Microscopic examination of vaginal fluid was performed in 17% of patients, assessment of vaginal pH in 15%, and whiff test in 21% (13). In the real-world setting, clinicians are moving away from microscopy, and alternative diagnostic methods are needed. Forty percent of the women in that study were prescribed inappropriate treatments for their symptoms of vaginitis (13). Interestingly, the women who were treated empirically for vaginitis were significantly more likely to return in the next 3 months than women who were not treated, suggesting that empirical treatment in women with no infectious cause identified may result in more symptom-triggered visits (13). This highlights the importance of considering multiple etiologies of the disease in patients, as well as the need for precise diagnosis of all infectious vaginitis/vaginosis, to ensure appropriate diagnosis and timely treatment both for the health of the patient and antimicrobial stewardship. MVP is able to differentiate the azole-resistant C. glabrata and C. krusei from each other more easily than treated *Candida* species. MVP also detected co-infections in 17.0% and 17.9% of CVS and SVS specimens, respectively, which is similar to rates found in other studies (13, 14). Most notably, MVP performed similarly in a laboratory-based setting by trained operators and in the POC setting by untrained operators, and, consequently, has the potential to be a valuable tool in a clinic setting for providing prompt and accurate results.

Several studies have demonstrated that the detection of Atopobium vaginae, BVAB2, and *Megasphaera*-1, either alone or, particularly, in combination, provided high diagnostic sensitivity for BV detection (24–27). MVP does not use Gardnerella vaginalis or *Lactobacillus* species in the algorithm that determines BV results. Since these 2 organisms can be found in patients with and without BV, comparator assays using these targets may potentially overcall the diagnosis of BV, thus affecting the observed accuracy of MVP by limitations in the performance of the comparator assays. Given the limitations of the standard methods for BV diagnosis that are currently used in clinical practice, MVP offers high potential for more accurate BV diagnosis in both laboratory and POC settings.

The Xpert Xpress MVP test was both highly sensitive and specific for the diagnosis of the most common causes of vaginitis/vaginosis, all from the same vaginal swab specimen, and can be used in a variety of settings, providing results in less than an hour. This test can be a valuable tool in the accurate, timely diagnosis and treatment of infectious vaginitis/vaginosis. This study demonstrates that MVP has the potential to be used in a POC setting to offer the opportunity for women to be diagnosed and treated, all within the same office visit, which could reduce the number of return visits and potentially lessen the adverse health outcomes for women with vaginitis/vaginosis.
